# Content of Vitamin D_2_ in Alternative Biological and Nutritional Sources and Its Effectiveness as Compared to Vitamin D_3_—A Narrative Review

**DOI:** 10.3390/metabo16070485

**Published:** 2026-07-10

**Authors:** Filip Bieg, Agnieszka Galanty, Patricia Arancibia-Ávila, Shela Gorinstein, Paweł Paśko

**Affiliations:** 1 Department of Food Chemistry and Nutrition, Faculty of Pharmacy, Medical College, Jagiellonian University, 30-688 Kraków, Poland; fibieg01@gmail.com; 2 Department of Pharmacognosy, Faculty of Pharmacy, Medical College, Jagiellonian University, 30-688 Kraków, Poland; agnieszka.galanty@uj.edu.pl; 3 Laboratory of Ecophysiology and Microalgae, Department of Basic Sciences, Faculty of Science, University of Bío-Bío, Chillán 3780247, Chile; parancib@ubiobio.cl; 4 Institute for Drug Research, School of Pharmacy, Faculty of Medicine, The Hebrew University of Jerusalem, Jerusalem 9112001, Israel; shela.gorin@mail.huji.ac.il

**Keywords:** vitamin D_2_, ergocalciferol, mushrooms, vitamin D_2_ pharmaceutical formulation, algae, vitamin D_2_ dietary supplements, pharmacy and food science

## Abstract

**Background/Objectives**: Interest in alternative, non-animal sources of vitamin D has increased due to the global prevalence of its deficiency and the growing demand for plant-based dietary options. Mushrooms and algae have emerged as potential sustainable sources of vitamin D_2_ and, in selected cases, vitamin D_3_ following ultraviolet (UV) exposure. However, the comparative bioavailability and clinical effectiveness of vitamin D_2_ relative to vitamin D_3_ remain controversial. This review aims to evaluate the content of vitamin D_2_ in mushrooms and algae, the impact of UV irradiation on its synthesis, and the effectiveness of vitamin D_2_ supplementation compared with vitamin D_3_ in humans. **Methods:** PubMed, ScienceDirect, and Google Scholar were searched (1996–2026). Only human intervention studies were included when assessing clinical efficacy. Data on natural sources and pharmaceutical formulations were analyzed. **Results:** UV irradiation markedly increases vitamin D_2_ content in mushrooms, as compared to cultivated products. Algal vitamin D content varies, depending on species and UV exposure, with no robust clinical trials confirming improvement of serum 25(OH)D after algal supplementation. Across multiple randomized controlled trials, vitamin D_2_ consistently increased circulating 25(OH)D_2_ but frequently reduced 25(OH)D_3_ and demonstrated lower efficacy in raising total 25(OH)D compared with vitamin D_3_, as confirmed by a recent meta-analysis. **Conclusions:** Although UV-enhanced mushrooms represent a quantifiable dietary source of vitamin D_2_, clinical evidence consistently indicates lower efficacy of vitamin D_2_ compared with vitamin D_3_. Algae cannot currently be considered a validated source of vitamin D for improving human vitamin D status. Further mechanistic and long-term clinical studies are required.

## 1. Introduction

Interest in vitamin D in modern pharmacy and dietetics has grown considerably in recent years. This trend is driven not only by the ongoing search for natural sources of vitamin D but also by the rising global prevalence of vitamin D deficiency, which remains a major public health concern affecting populations across different age groups and geographic regions [1,2]. At the same time, the expanding population of vegans and vegetarians increasingly expects new vitamin D-rich non-animal sources. This group has become particularly sensitive to nutritional deficiencies of compounds that are not normally present in edible plant-based foods. These include vitamin D_3_ and vitamin B_12_, as well as nutrients that are present in lower amounts (e.g., iodine), which may have long-term health consequences if not adequately supplemented or monitored [3,4].

**Figure 1:**
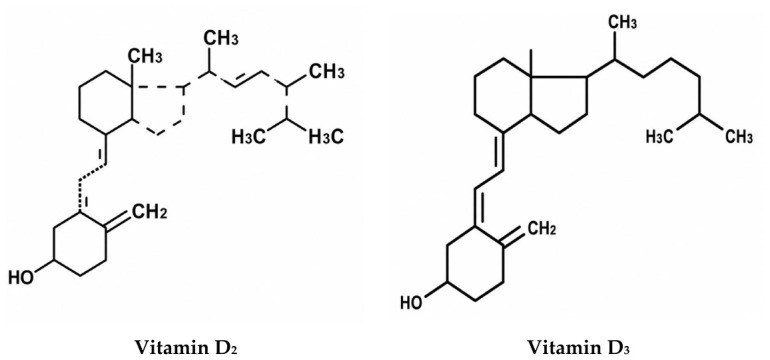
Chemical structure of ergocalciferol (Vitamin D_2_) and cholecalciferol (Vitamin D_3_).

Vitamin D plays a crucial role in maintaining calcium and phosphate homeostasis, thereby supporting bone mineralization and preventing disorders such as rickets, osteomalacia, and osteoporosis. Beyond skeletal health, it modulates immune function, regulates cell proliferation and differentiation, and has been implicated in the prevention of autoimmune diseases, infections, and certain cancers [5]. Among the vitamin D group, vitamin D3 (cholecalciferol) and D2 (ergocalciferol) are the most important from the perspective of human health. Structural differences (Figure 1) between these two vitamins may influence their affinity for vitamin D-binding protein, metabolic stability, and circulating half-life, which in turn can affect their biological activity and clinical effectiveness [6,7].

Traditionally, vitamin D_3_ has been derived primarily from animal-based foods such as fatty marine fish (e.g., salmon, mackerel, herring, and sardines), cod liver oil and other fish oils, fish and animal liver, egg yolk, butter, and full-fat dairy products. In addition, vitaminD_3_-fortified products such as milk, plant-based beverages, margarines and other spreadable fats as vegetable oils, and breakfast cereals are also available [8]. In contrast, growing attention has been directed toward expanding the range of foods that can naturally provide vitamin D_2_ or produce vitamin D_3_ following ultraviolet (UV) exposure. Mushrooms and algae are particularly promising in this context and are increasingly recognized as potential components of functional and sustainable dietary patterns [9].

Despite decades of research, the relative efficacy of vitamin D_2_ compared with vitamin D_3_ remains a subject of scientific debate. Therefore, the aim of this review is to comprehensively evaluate the actual content of vitamin D_2_ in alternative natural sources, i.e., mushrooms, algae, and microalgae, also in terms of the enhancement of its synthesis following UV irradiation. Additionally, this review focuses on critical analysis of the bioavailability and effectiveness of vitamin D_2_ supplementation, as compared to vitamin D_3_, based on the existing evidence from human studies.

## 2. Materials and Methods

An extensive literature search was performed to identify relevant publications concerning vitamin D_2_ and vitamin D_3_ in biological, nutritional, pharmaceutical, and clinical contexts. The review was designed as a narrative review based on a structured search strategy. The search covered scientific literature published in English between 1996 and 2026, and was conducted using PubMed, ScienceDirect, and Google Scholar. These databases were selected to provide broad coverage of biomedical, nutritional, food science, biochemical, analytical, and clinical studies relevant to vitamin D_2_ sources, metabolism, bioavailability, potency, and stability.

The literature search was structured according to two main objectives of the review. The first objective was to identify biological and nutritional sources of vitamin D_2_ and vitamin D_3_, particularly edible mushrooms, macroalgae, and microalgae, and to summarize reported concentrations, UV-mediated formation, and stability in different matrices. The second objective was to compare vitamin D_2_ and vitamin D_3_ in terms of metabolism, bioavailability, potency, stability, and effectiveness in human studies when delivered as medicinal products, dietary supplements, or vitamin D-rich food products.

For the first objective, the following search strings were applied: (“vitamin D_2_” OR ergocalciferol) AND (source OR occurrence OR concentration OR content OR level) AND (mushroom* OR fungi OR yeast OR algae OR seaweed OR microalgae OR lichen OR “fortified food*” OR “fermented food*” OR “plant-based food*”) (“vitamin D_2_” OR ergocalciferol) AND (ergosterol OR “provitamin D_2_”) AND (ultraviolet OR UV OR UVB OR irradiation OR “UV irradiation” OR “UV-B exposure”)

For the second objective, the following search strings were applied: (“vitamin D_2_” OR ergocalciferol) AND (“vitamin D_3_” OR cholecalciferol) AND (metabolism OR bioavailability OR potency OR efficacy OR stability OR degradation) (“vitamin D_2_” OR ergocalciferol) AND (“vitamin D_3_” OR cholecalciferol) AND (“25-hydroxyvitamin D” OR “25(OH)D” OR “vitamin D status” OR supplementation OR trial OR intervention).

**Figure 2:**
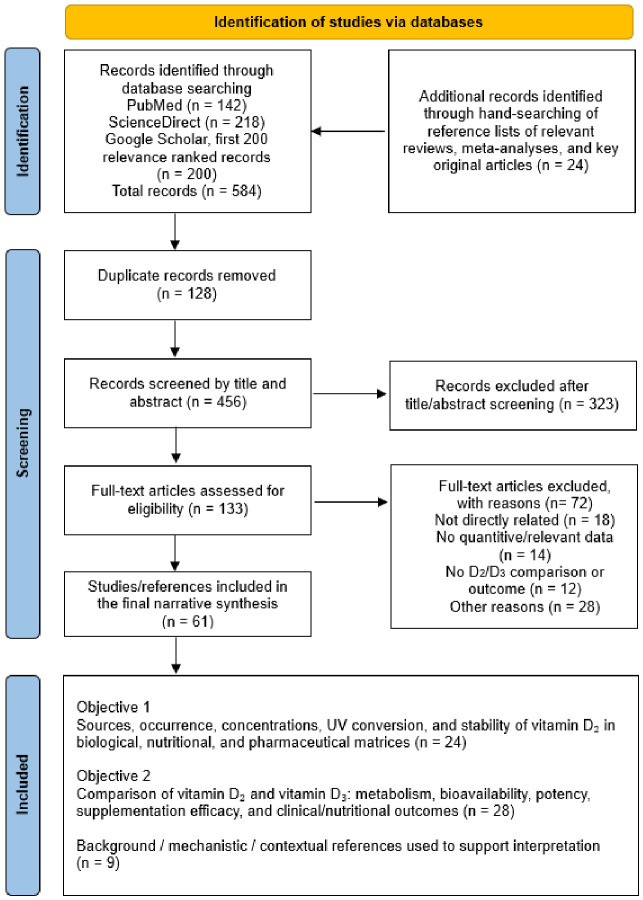
PRISMA-style flow diagram of the literature identification and selection process.

The study selection process was performed in several stages, presented in Figure 2. For the first objective, eligible publications included analytical, nutritional, food composition, technological, and experimental studies reporting vitamin D_2_ or vitamin D_3_ content in edible mushrooms, macroalgae, microalgae, or related biological and nutritional matrices. Studies addressing ergosterol conversion, UV exposure, irradiation conditions, processing, storage, or stability of vitamin D_2_ were also considered eligible.

For the second objective, eligible publications included human intervention studies, randomized or controlled trials, feeding studies, and clinically relevant studies comparing vitamin D_2_ and vitamin D_3_ or evaluating the effect of vitamin D_2_ supplementation on serum 25(OH)D_2_, 25(OH)D_3_, and total 25(OH)D concentrations. Studies involving vitamin D_2_ delivered as medicinal products, dietary supplements, fortified foods, or vitamin D_2_-rich food products were included when they provided relevant information on bioavailability, potency, or effectiveness. In vitro, in silico, and animal studies were excluded as not relevant. However, mechanistic and biochemical studies were considered where necessary to support interpretation of differences in metabolism, transport, hydroxylation, catabolism, and stability between vitamin D_2_ and vitamin D_3_.

Articles were excluded if they were not directly related to vitamin D_2_, vitamin D_3_, or ergosterol-derived vitamin D formation; did not provide relevant information on vitamin D content, metabolism, bioavailability, potency, stability, or clinical/nutritional effectiveness; lacked sufficient methodological or analytical detail; or were not available in full text. Reviews, systematic reviews, and meta-analyses were used mainly to identify additional primary studies and to support the interpretation of the evidence, but they were not treated as primary evidence in the tabulated synthesis unless directly relevant to the narrative discussion.

The complete identification and selection process, including the number of records identified, duplicates removed, records screened, full-text articles assessed, excluded articles, and studies included in the final synthesis, is summarized in the PRISMA-style flow diagram presented in Figure 2.

## 3. Results

### 3.1. Vitamin D and Edible Mushrooms

Edible mushrooms are increasingly recognized as one of the few non-animal dietary sources of vitamin D, specifically vitamin D_2_. Unlike plants, fungi contain substantial amounts of ergosterol in their cell membranes, where it plays a structural role, analogous to cholesterol in animals, contributing to membrane integrity, fluidity, and intracellular transport [10,11]. Upon exposure to ultraviolet (UV) radiation, ergosterol undergoes photochemical conversion to pre-vitamin D_2_, which is subsequently thermally isomerized to ergocalciferol (vitamin D_2_). No data exists on the ability of edible mushrooms to synthesize vitamin D_3_.

#### 3.1.1. Content of Vitamin D_2_

Wild mushrooms exposed naturally to sunlight may contain nutritionally significant amounts of vitamin D_2_ (Table 1). Finnish funnel chanterelles (*Cantharellus tubaeformis*) collected in late summer and early autumn were reported to contain between 3 and 30 µg vitamin D_2_ per 100 g fresh weight (FW), whereas retail button mushrooms (*Agaricus bisporus*) typically contained less than 1 µg/100 g FW [12]. Subsequent analyses identified high vitamin D_2_ levels in other wild species, including *Boletus edulis* (up to 58.7 µg/100 g FW) and *Cantharellus cibarius* (10.7 µg/100 g FW) [13]. These findings highlight the strong influence of natural UV exposure on vitamin D_2_ accumulation in wild-harvested mushrooms. Commercially cultivated mushrooms are generally grown in controlled environments without sunlight exposure. As a result, their vitamin D_2_ content is usually negligible (<1 µg/100 g FW), as documented for button, shiitake, and oyster mushrooms in multiple food composition databases [11,14].

#### 3.1.2. Influence of UV Exposure to Enhance Vitamin D_2_ Content

Intentional exposure to UV radiation markedly enhances vitamin D_2_ content. Controlled exposure of fresh mushrooms to midday sunlight for 15–60 min has been shown to increase vitamin D_2_ concentrations to 10–30 µg/100 g FW, approaching or meeting daily intake recommendations in many countries [10,13]. Jasinghe & Perera’s [15] analysis of ergosterol content in different tissues of Shiitake mushrooms showed a significant difference in its distribution, with the highest content found in button mushrooms (7.80 ± 0.35 mg/g DM) and the lowest in enoki mushrooms (0.68 ± 0.14 mg/g DM). The conversion of ergosterol to vitamin D_2_ was about four times higher for the gills exposed to UV-A irradiation than the outer caps. The lowest conversion of ergosterol to vitamin D_2_ (12.5 ± 0.28 μg/g DM) was observed for button mushrooms, while the highest (45.1 ± 3.07 μg/g DM) was observed for oyster mushrooms. Even higher concentrations can be achieved under controlled UV-B lamp irradiation. Post-harvest UV-B treatment has produced vitamin D_2_ concentrations ranging from 40 to over 200 µg/g DM, depending on the exposure time, irradiance intensity, temperature, moisture content, and mushroom morphology [11]. Pulsed UV systems have been demonstrated to be particularly efficient. Short exposures (few seconds) using high-energy pulsed UV lamps can generate nutritionally relevant vitamin D_2_ levels comparable to those achieved with longer fluorescent UV exposure [11,16]. Huang et al. [17] evaluated eleven species of fresh mushroom fruiting bodies, including species from six genera, *Agaricus*, *Agrocybe*, *Auricularia*, *Hypsizigus*, *Lentinula* and *Pholiota*, and five species from the *Pleurotus* genus, irradiated with UV-B light for 2 h. For three species of mushroom fruiting bodies with excellent vitamin D_2_ yield, their mycelia were obtained by liquid culture and subjected to the same UV-B irradiation. Vitamin D_2_ content of irradiated fruiting bodies significantly increased from 0–3.9 to 15.1–208.6 μg/g, of which the amount in golden oyster mushroom increased by a maximum of 204.7 μg/g. Vitamin D_2_ content in irradiated mycelia of golden oyster, oyster and pink oyster mushrooms increased from 0.3–5.9 to 66–82 μg/g, respectively. Freeze-drying combined with UV irradiation may further enhance conversion efficiency due to improved UV penetration into porous tissue structures [10].

**Table 1:** Reported vitamin D_2_ content of edible mushrooms across the reviewed studies.

Mushroom Species/Type	Country	Treatment/Condition	Vitamin D_2_ Content	Reference
Funnel chanterelle (*Cantharellus tubaeformis*)	Finland	Wild, natural sunlight (late summer/early autumn)	3–30 µg/100 g FW	[12]
Button mushroom (*Agaricus bisporus*)	Finland	Retail, no UV exposure	<1 µg/100 g FW	[12]
Cep (*Boletus edulis*)	Sweden	Wild	Up to 58.7 µg/100 g FW	[13]
Chanterelle (*Cantharellus cibarius*)	Sweden	Wild	10.7 µg/100 g FW	[13]
Button, shiitake (*Lentinula edodes)*, oyster (*Pleurotus ostreatus*) mushrooms	Australia	Commercially cultivated, no sunlight	<1 µg/100 g FW (negligible)	[11,14]
Fresh mushrooms (general)	Singapore/Sweden	Midday sunlight, 15–60 min	10–30 µg/100 g FW	[10,13]
Button mushroom	Singapore	UV-A irradiation (315–400 nm), 3.5 W/m^2^ at 15 cm, 2 h (dose 25.2 kJ/m^2^), 27 °C, 65% RH; lowest conversion	12.5 ± 0.28 µg/g DM	[15]
Oyster mushroom	Singapore	UV-A irradiation (315–400 nm), 3.5 W/m^2^ at 15 cm, 2 h (dose 25.2 kJ/m^2^), 27 °C, 65% RH; highest conversion	45.1 ± 3.07 µg/g DM	[15]
Mushrooms (general)	Australia	Post-harvest UV-B lamp treatment, UV-B (280–315 nm); e.g., 1.14 W/m^2^, 90 min, 28 °C	40 to >200 µg/g DM	[11]
Fruiting bodies, 11 species (6 genera)	Taiwan	UV-B (280–360 nm), 0.36 mW/cm^2^ at 19 cm, 2 h (dose 25.9 kJ/m^2^), 25 °C, fresh/moist (87–90% moisture)	From 0–3.9 to 15.1–208.6 µg/g	[17]
Golden oyster mushroom (fruiting body)	Taiwan	UV-B (280–360 nm), 0.36 mW/cm^2^ at 19 cm, 2 h (dose 25.9 kJ/m^2^), 25 °C, fresh/moist (87–90% moisture) (maximum increase)	+204.7 µg/g (increase)	[17]
Golden oyster, oyster, pink oyster (mycelia)	Taiwan	UV-B (280–360 nm), 0.36 mW/cm^2^ at 19 cm, 2 h (dose 25.9 kJ/m^2^), 25 °C, fresh/moist (87–90% moisture)	From 0.3–5.9 to 66–82 µg/g	[17]

FW, fresh weight; DM, dry matter; RH, relative humidity.

#### 3.1.3. Influence of Technological and Culinary Processes on Vitamin D_2_ Content

The stability of vitamin D_2_ in UV-enhanced mushrooms during storage has been examined under refrigeration and dry storage conditions. Fresh UV-exposed mushrooms stored at 2–4 °C generally retain nutritionally relevant amounts of vitamin D_2_ for up to one week, although some studies report gradual declines following first-order kinetics. In contrast, other investigations observed minimal degradation over 7–14 days of refrigerated storage [18,19,20]. Variability likely reflects differences in moisture content, packaging, and initial vitamin D_2_ concentration. In dried mushrooms stored in dark, dry conditions at room temperature, vitamin D_2_ retention remains relatively high for up to eight months, followed by gradual losses during prolonged storage [11]. Nevertheless, even after extended storage, concentration may remain nutritionally meaningful. Thermal processing can influence vitamin D_2_ retention, although losses are generally moderate. Frying without oil for short durations (approximately 5 min) preserves 85–88% of vitamin D_2_ content after adjusting for moisture loss. Boiling and oven-baking results in somewhat greater losses, with retention rates of approximately 60% [21,22]. The method and duration of cooking appear to be critical determinants of vitamin D_2_ preservation (Table 2).

### 3.2. Vitamin D and Algae

Macro- and microalgae represent diverse groups of photosynthetic organisms classified primarily according to their size and structural organization—macroalgae are multicellular and visible to the naked eye (e.g., wakame *Undaria pinnatifida*, kombu *Saccharina japonica*, nori *Pyropia* spp., e.g., *Pyropia yezoensis*), whereas microalgae are microscopic and typically unicellular (e.g., *Chlorella*, *Spirulina*, *Dunaliella*). Both groups started to play an important role in nutrition and medicine.

Evidence regarding the occurrence and synthesis of vitamin D in algae remains limited and heterogeneous. Reported concentrations of vitamin D_2_ and vitamin D_3_ in microalgae vary considerably across studies, likely reflecting differences in taxonomy, environmental exposure (particularly UV radiation), analytical methodology, and seasonal variability. The significant determinant appears to be the sterol composition of algal membranes, as vitamin D formation depends on the presence of specific sterol precursors. In biological systems, vitamin D is generated via a non-enzymatic photochemical reaction triggered by UV-B radiation, as described previously (ergosterol into vitamin D_2_), but in the case of some algae, 7-dehydrocholesterol (7-DHC) may be converted into vitamin D_3_. This transformation involves photoconversion to previtamin D followed by thermal isomerization. As emphasized by Göring [9], vitamin D in natural systems may represent either a physiologically relevant metabolite or a photochemical by-product of membrane sterol degradation. Algae exhibit extraordinary sterol diversity [23] with clear taxonomic trends: (i) *Rhodophyta* (red algae): predominantly cholesterol; some species rich in desmosterol; (ii) *Phaeophyta* (brown algae): fucosterol as the dominant sterol; (iii) *Chlorophyta* (green algae): highly variable; contain 24-ethyl sterols, cholesterol, and in some species ergosterol. This biochemical heterogeneity complicates generalizations regarding algal vitamin D production.

#### 3.2.1. Synthesis and Content of Vitamin D

Several studies have reported measurable amounts of vitamin D in micro- and macroalgae [24,25,26,27,28,29,30], with the available quantitative data summarized in Table 3. However, the reported concentrations vary considerably, and in some studies vitamin D was not detected or was present below the limit of detection or quantification. These discrepancies may reflect differences in algal species, cultivation and environmental conditions, sample processing, and analytical methodology, and they indicate that the occurrence and nutritional relevance of vitamin D in algae require further systematic investigation. Rao and Raghuramulu [24] reported elevated levels of ergosterol (390 µg/100 g dw), 7-DHC (2400 µg/100 g dw), vitamin D_2_ (5.3 µg/100 g dw), and vitamin D_3_ (80 µg/100 g dw) in freshwater microalgae consisted of green (*Pediastrum*, *Scenedesmus*, *Crucigenia*, *Coelastrum*, *Chlorella*, and *Cosmarium*), blue green (*Gomphosphaeria* and *Oscillatoria*), and brown (*Synedra*, *Navicula*, and *Cyclotella*) algae. In contrast, Brown et al. [25] found vitamin D_2_ and D_3_ concentrations below the detection limit (≤0.45 μg/g) in four Australian microalgae species (*Nannochloropsis*-like sp., *Pavlova pinguis*, *Stichococcus* sp., and *Tetraselmis* sp.). These discrepancies likely arise from differences in analytical sensitivity (e.g., HPLC vs. earlier methods), seasonal UV exposure, cultivation conditions, and intrinsic species variability.

#### 3.2.2. Influence of UV Irradiation on Vitamin D Content

Experimental evidence shows that UV-B exposure can stimulate vitamin D_3_ production in selected microalgae, supporting a sunlight-dependent mechanism. Of the four species, *Nannochloropsis oceanica* was able to produce vitamin D_3_ (up to 1 ± 0.3 µg/g DM), and the production was significantly enhanced by increasing the dose of the UVB. In contrast, *Chlorella minutissima*, *Arthrospira maxima* and *Rhodomonas salina* were not able to produce vitamin D_3_. These findings suggest that *N. oceanica* exposed to artificial UVB could be used as a new natural source of vitamin D_3_ [26]. Schneider et al. [27] evaluated the induction of vitamin D_3_ synthesis in different algae after UV radiation and found that its highest levels of synthesis were found in *Porphyra umbilicalis*, *Sargassum vulgare*, and *Ulva lactuca*.

Recent investigations in vitamin D metabolism in microalgae, particularly in the coccolithophore *Emiliania huxleyi*, a globally abundant marine species, revealed that the species contains ergosterol and 7-DHC, enabling potential synthesis of both vitamin D_2_ and D_3_. Eliason et al. [28] demonstrated that UV-B exposure stimulates vitamin D production in *E. huxleyi* and showed that vitamin D_2_ was significantly increased in UV-exposed cultures, with levels of approximately ∼4 ng/mg DM, while it was barely detected in cultures that were not exposed to UV. The D_2_ precursor ergosterol was found in both UV-treated and control cultures. Lower amounts of vitamin D_3_ (~0.04 ng/mg DM) were detected in both UV-treated and control cultures.

Claims regarding vitamin D_3_ in green macroalgae, particularly *Ulva* species, are increasingly common in non-scientific sources. Analytical studies using HPLC have detected vitamin D_3_ in some *Ulva* samples; however, concentrations often increase following UV exposure, indicating photochemical formation rather than constitutive biosynthesis [29]. Hughes et al. [30], who used a highly sensitive analytical method (LC-QQQ), noted very low levels of vitamin D_2_ and D_3_ and their hydroxy derivatives in Australian wakame, and kombu.

At present, definitive evidence supporting a conserved and regulated vitamin D metabolic pathway in algae remains incomplete. The biological significance of vitamin D in these organisms, as well as their potential application in human nutrition and pharmaceutical development, therefore, remains an open question. As recently emphasized by several authors, analytical methods used to quantify vitamin D_2_ and vitamin D_3_ in micro- and macroalgae must be highly sensitive and selective, since these compounds may co-elute or interfere with chlorophylls and other lipophilic pigments [30]. The reliable quantification of vitamin D in algae is further challenged by its low concentrations and the inconsistent detection of vitamin D_3_, which has prompted the use of chromatographic separation coupled with tandem mass spectrometry to improve sensitivity and selectivity [28]. Further research integrating sterol profiling, controlled UV-exposure experiments, transcriptomic analyses, and metabolomic approaches is necessary to determine whether vitamin D production in algae represents an evolutionary adaptation or merely incidental photochemical transformation. Table 3 summarizes the reported vitamin D_2_ and vitamin D_3_ contents of selected micro- and macroalgae across the cited studies.

### 3.3. Pharmaceutical Products and Dietary Supplements Containing Vitamin D_2_

Vitamin D_2_ is available as medicines and dietary supplements. Prescription-only medicines are indicated mainly for the treatment of confirmed vitamin D deficiency. These products are marketed in several pharmaceutical forms, including oral tablets and capsules, oil-based oral solutions, and intermittent high-dose formulations, most commonly providing single doses in the range of 20,000–50,000 IU. Interestingly, vitamin D_2_ (300,000 IU) is also available in solution for injection, as high-dose parenteral formulation intended for the rapid correction of severe vitamin D deficiency, typically administered via the intramuscular route. This formulation bypasses gastrointestinal absorption and is therefore particularly suitable for patients with malabsorption syndromes, severe deficiency states, or poor adherence to oral therapy. The injectable form provides a depot effect, releasing ergocalciferol slowly over time [31]. It should be emphasized that vitamin D_2_ is also available in multivitamin and multimineral combinations designed for daily supplementation rather than therapeutic correction of deficiency. The presence of dietary fats and minerals in the capsule matrix supports absorption; however, the dose of vitamin D_2_ is relatively low and intended to contribute to maintenance of vitamin D status as part of overall nutritional support [32]. Oral liquid multivitamins in which ergocalciferol is included as vitamin D are specifically designed for populations with swallowing difficulties, including pediatric patients and individuals requiring enteral feeding. The product is commonly used in clinical settings such as metabolic disorders, restrictive diets (e.g., ketogenic diets), or conditions associated with limited nutritional intake. As with other oral D_2_ formulations, the pharmacokinetic profile is characterized by a shorter duration of action compared with vitamin D_3_, which is acknowledged in clinical guidance and reflected in dosing recommendations [33].

Beyond medicinal products, vitamin D_2_ is also marketed as a dietary supplement, targeting individuals who avoid animal-derived ingredients. The source of vitamin D_2_ used in supplements is non-animal and includes UV-irradiated mushrooms, fermented yeast, or laboratory synthesis from ergosterol. Such supplements are commonly available as capsules or tablets frequently labeled as suitable for vegan diets. Typical doses in dietary supplements range from 400 to 2000 IU per day, which aligns with nutritional supplementation.

### 3.4. Effectiveness and Bioavailability of Vitamin D_2_ in Comparison to Vitamin D_3_

To facilitate comparison of the available human evidence, the main characteristics of intervention studies comparing vitamin D_2_ and vitamin D_3_, including study design, population, intervention, analytical method, and principal outcomes, are summarized in Table 4.

#### 3.4.1. Daily Treatment of Vitamin D_2_

- 200–400 IU

Nimitphong et al. [34] investigated whether genetic variation in vitamin D-binding protein (DBP, rs4588) affects serum 25-hydroxyvitamin D [25(OH)D] response to vitamin D supplementation. Thirty-nine healthy adults received 400 IU/day of either vitamin D_3_ or D_2_ for three months. Vitamin D_3_ supplementation significantly increased 25(OH)D_3_ (+16.2 ± 4.2 nmol/L), whereas vitamin D_2_ raised 25(OH)D_2_ (+22.0 ± 2.1 nmol/L) but reduced 25(OH)D_3_ (−14.2 ± 2.0 nmol/L). After three months, total 25(OH)D tended to be higher with vitamin D_3_ than D_2_ (67.8 ± 3.9 vs. 61.0 ± 3.0 nmol/L). Individuals carrying CA or AA alleles showed a significantly smaller increase in total 25(OH)D and 25(OH)D_3_ compared with CC homozygotes following vitamin D_3_ supplementation, while no genotype-related differences were observed with vitamin D_2_. These findings indicate that DBP rs4588 polymorphism influences responsiveness to vitamin D_3_, but not vitamin D_2_ supplementation.

An interesting study was conducted by Gallo et al. [35], who evaluated healthy breast-fed infants (*n* = 52) receiving 400 IU/day of D_2_ or D_3_ for 3 months. The increase in total 25(OH)D did not differ between groups (D_2_: 17.6 ± 26.7 nmol/L; D_3_: 22.2 ± 20.2 nmol/L). Nevertheless, 75% of infants in the D_2_ group vs. 96% in the D_3_ group achieved ≥50 nmol/L (*p* < 0.05). The final conclusion highlighted that both forms are acceptable in early infancy, although D_3_ may improve attainment of sufficiency thresholds.

- 1000 IU

Randomized, double-blind, placebo-controlled trial assessed whether vitamin D_2_ is as effective as vitamin D_3_ in maintaining serum 25-hydroxyvitamin D in healthy adults aged 18–84 years supplemented for 11 weeks at the end of winter. At baseline, 60% of participants were vitamin D-deficient. Serum 25(OH)D increased similarly with 1000 IU/day of vitamin D_2_ (16.9 ± 10.5 to 26.8 ± 9.6 ng/mL), vitamin D_3_ (19.6 ± 11.1 to 28.9 ± 11.0 ng/mL), and a combined dose of 500 IU D_2_ plus 500 IU D_3_ (20.2 ± 10.4 to 28.4 ± 7.7 ng/mL). Importantly, daily vitamin D_2_ did not reduce circulating 25(OH)D_3_ concentrations; however, neither form raised levels above 30 ng/mL in deficient individuals. These findings indicate that vitamin D_2_ is comparable to vitamin D_3_ for maintaining vitamin D status at a dose of 1000 IU/day [36].

Biancuzzo et al. [37], in a double-blind placebo-controlled study, evaluated thirty-four adults receiving 1000 IU/day of D_2_ or D_3_ for 11 weeks. Both forms raised total 25(OH)D to a similar extent. Vitamin D_2_ increased 1,25(OH)_2_D_2_ by 7.4 pg/mL but caused a 9.9 pg/mL decrease in 1,25(OH)_2_D_3_, leaving total active vitamin D unchanged. Both isoform maintained total vitamin D, although D_2_ shifted the balance between active metabolites.

In another randomized, double-blind trial, 95 hip fracture in patients with vitamin D insufficiency (<50 nmol/L) received 1000 IU/day of either vitamin D_2_ or D_3_ for three months. Among the 70 participants who completed the study, vitamin D_3_ produced a significantly greater increase in serum 25OHD than D_2_ (31% higher by HPLC and 52% higher by RIA), while changes in parathyroid hormone (PTH) levels did not differ between groups. These findings indicate that vitamin D_3_ is more effective at improving vitamin D status in this high-risk population, although the clinical relevance remains uncertain due to similar PTH responses [38].

- 2000 IU

Lehmann et al. [39] in the double-blind randomized trial evaluated supplementation with 50 µg/day (2000 IU) of vitamin D_2_ or D_3_ for 8 weeks in 107 adults, with measurements at baseline, 4, and 8 weeks. After 8 weeks, total 25(OH)D increased by +30.2 ± 20.1 nmol/L with D_2_ vs. +45.5 ± 21.7 nmol/L with D_3_ (*p* = 0.001). 25(OH)D_3_ decreased by −19.8 nmol/L in the D_2_ group but increased by +46.5 nmol/L in the D_3_ group (*p* = 0.001). The major explanation for D_3_ superiority was a substantial decline in endogenous 25(OH)D_3_ after D_2_ supplementation, despite similar hydroxylation of both vitamins. It was concluded that vitamin D_3_ raises vitamin D status more effectively than D_2_; the reduction in 25(OH)D_3_ during D_2_ therapy questions the usefulness of D_2_ for supplementation.

- 4000 IU

A sole study compared the efficacy of equal doses of vitamins D_2_ and D_3_ in increasing serum 25-hydroxyvitamin D among healthy adults (*n* = 72), including 17 participants receiving vitamin D_2_ and 55 receiving vitamin D_3_. Subjects were supplemented with 4000 IU daily for 14 days, and total 25(OH)D was measured using an assay detecting both forms. Vitamin D_3_ produced a significantly greater rise in 25(OH)D than vitamin D_2_ (23.3 vs. 13.7 nmol/L), corresponding to approximately 1.7-fold higher efficacy, with the strongest responses observed in individuals with lower baseline vitamin D levels. These results question the presumed equivalence of vitamins D_2_ and D_3_ and indicate that D_3_ is more effective for improving vitamin D status [40].

#### 3.4.2. Weekly Treatment of Vitamin D_2_

Heaney et al. [41], in a single-blind randomized trial, compared the potency of vitamins D_2_ and D_3_ in 33 healthy adults receiving 50,000 IU weekly for 12 weeks. Vitamin D_3_ produced a significantly greater increase in serum 25(OH)D than D_2_ (area under the curve: 2136 vs. 1366 ng·d/mL; steady-state increments: 45 vs. 24 ng/mL) and resulted in substantially higher accumulation in subcutaneous fat. Overall, D_3_ was estimated to be about 87% more potent than D_2_ in raising and maintaining vitamin D status, supporting its preferential use in the treatment of deficiency.

#### 3.4.3. Monthly Treatment of Vitamin D_2_

In a sole randomized clinical trial, 64 community-dwelling adults aged ≥65 years received vitamin D_2_ or D_3_ either daily (1600 IU) or once monthly (50,000 IU) for 12 months to compare dosing strategies. At baseline, 40% of participants had serum 25(OH)D levels below 30 ng/mL; after one year, deficiency persisted in 19% (*n* = 12; seven in the daily group and five in the monthly group) despite adherence exceeding 91%. Both regimens significantly increased circulating 25(OH)D without altering serum calcium, 24 h urinary calcium, parathyroid hormone, or bone turnover markers, and the highest observed concentration reached 72.5 ng/mL. Vitamin D_3_ was slightly but significantly more effective than D_2_, whereas D_2_ supplementation increased 25(OH)D_2_ but significantly reduced 25(OH)D_3_. Overall, the findings indicate that both daily and monthly dosing safely improves vitamin D status, although responses vary considerably between individuals [42].

#### 3.4.4. Single High Dose of Vitamin D Administered Orally or Intramuscularly

Hammami et al. [43], in two blinded randomized studies with approximately 100 volunteers receiving 50,000 IU of vitamin D_2_ or D_3_, monitored serum metabolites for 56 days. Compared with placebo, vitamin D_2_ administration significantly reduced 25(OH)D_3_ by 13.2 nmol/L at day 28 and 10.8 nmol/L at day 56, whereas vitamin D_3_ supplementation produced a comparable but shorter-lived decrease in 25(OH)D_2_ (9.8 nmol/L at day 28; 1.7 nmol/L at day 56). Strong inverse correlations between changes in the two metabolites suggest a common regulatory process governing vitamin D metabolism. These findings indicate that reciprocal reductions in 25(OH)D_2_ and 25(OH)D_3_ are likely driven by the overall increases in circulating vitamin D rather than being specific to either form. Relative efficacy is time-dependent and influenced by dosing frequency; in addition, daily dosing may mask the shorter half-life of D_2_. Romagnoli et al. [44], in a prospective randomized study, evaluated the potency of a single high dose (300,000 IU) of vitamins D_2_ and D_3_ administered orally or intramuscularly to 32 vitamin D-deficient elderly women (66–97 years) residing in a nursing home. Serum 25(OH)D increased rapidly by day 3 only after oral administration, with significantly higher levels at day 30 following oral vitamin D_3_ (47.8 ± 7.3 ng/mL), compared with intramuscular vitamin D_3_ (15.9 ± 11.3 ng/mL), oral vitamin D_2_ (17.3 ± 4.7 ng/mL), and intramuscular vitamin D_2_ (5.0 ± 4.4 ng/mL) administration. The area under the curve over 60 days confirmed greater efficacy of D_3_ versus D_2_ for both oral (3193 ± 759 vs. 1820 ± 512 ng·d/mL) and intramuscular routes (1361 ± 492 vs. 728 ± 195 ng·d/mL). Higher 25(OH)D concentrations were associated with significant reductions in parathyroid hormone at multiple time points, with cholecalciferol producing a stronger PTH-lowering effect by day 60. Vitamin D_3_ was nearly twice as potent as D_2_ regardless of the route of administration.

#### 3.4.5. Supplementation of Vitamin D-Fortified Products

Fisk et al. [45], in a double-blind randomized trial in 40 healthy adults, tested fortified milk containing 5 (200 IU) or 10 (400 IU) µg/day of D_2_ or D_3_ as malted in milk drink for 4 weeks. It should be highlighted that this study was conducted in period of minimal UV-B exposure in the United Kingdom. Both vitamins produced dose-dependent increases in their respective metabolites without significant differences between groups. At low dietary doses, D_2_ and D_3_ appear equipotent for increasing 25(OH)D.

Tripkovic et al. [46], in a double-blind, randomized, placebo-controlled trial, examined whether daily fortification with biscuits and juice enriched in vitamin D_2_ or vitamin D_3_ (15 µg/day; 600 IU) could improve wintertime vitamin D status in 335 healthy South Asian and non-Asian, European women aged 20–64 years. Serum total 25-hydroxyvitamin D [25(OH)D] concentrations were assessed at baseline, week 6, and week 12. When data from both ethnic groups were combined, fortification with vitamin D_2_ resulted in moderate but significant increases in total 25(OH)D, with rises of 33 and 34% in the juice and biscuit groups, respectively. In contrast, vitamin D_3_ fortification produced markedly greater effects, with increases of 75 and 74% in the corresponding juice and biscuit groups. Absolute changes from baseline were significantly higher in both vitamin D_3_ groups compared with vitamin D_2_ and placebo groups, while no differences were observed between juice and biscuit formats within the same vitamin D form, indicating equivalent bioavailability of the food vehicles. At the end of the 12-week intervention, all non-Asian European women receiving vitamin D_3_ achieved serum 25(OH)D concentrations above 50 nmol/L, compared with approximately 90% of those receiving vitamin D_2_. Among South Asian women—who exhibited lower baseline 25(OH)D concentrations (<30 nmol/L)—72.7% of participants receiving vitamin D_3_ reached the 50 nmol/L threshold, whereas only 55.6% of those receiving vitamin D_2_ achieved sufficiency. Notably, South Asian women consuming vitamin D_2_ did not, on average, reach 50 nmol/L, in contrast to those receiving vitamin D_3_. Analysis of vitamin D metabolites showed that vitamin D_2_ fortification led to a pronounced increase in serum 25(OH)D_2_ but was accompanied by the reduction in circulating 25(OH)D_3_. In contrast, vitamin D_3_ fortification resulted in substantial increases in 25(OH)D_3_ concentrations. These findings support mechanistic explanations involving competitive metabolism between vitamin D_2_ and D_3_, differences in affinity for vitamin D-binding protein, hepatic hydroxylation efficiency, and the shorter half-life of 25(OH)D_2_. The authors concluded that vitamin D_3_ is significantly more efficacious than vitamin D_2_ in improving and maintaining wintertime vitamin D status at nutritionally relevant doses, particularly in populations at higher risk of deficiency.

A double-blind, randomized feeding trial evaluated whether daily consumption of UV-treated white button mushrooms (*Agaricus bisporus*) increases serum vitamin D status in healthy adults, aged 20–59 years (*n*~40, ≈10 per group). Participants consumed one serving (½ cup; 87.9 g) of cooked mushrooms daily for 6 weeks. Two levels of UV-treated mushrooms were compared with vitamin D_2_ capsules and untreated mushrooms. The study aimed to determine whether the intake would: (i) increase serum 25-hydroxyergocalciferol [25(OH)D_2_], and (ii) improve total 25(OH)D, the marker of vitamin D status. The work demonstrates that food-based vitamin D_2_ can serve as a dietary strategy to improve vitamin D intake. No direct comparison with D_3_ efficacy was performed; therefore, relative potency cannot be inferred from this study [47].

In a separate, double-blind randomized placebo-controlled trial, including 90 healthy adults aged 40–65 years, the participants received either 15 µg/day of vitamin D_2_ from a commercially available, UV-enhanced button mushroom (*Agaricus bisporus*) powder or 15 µg/day of vitamin D_3_ in capsule form for four weeks. At baseline, over half of the cohort was vitamin D-deficient (<50 nmol/L). The intake of vitamin D_2_ resulted in a 128% increase in serum 25(OH)D_2_, rising from 3.9 ± 1.9 nmol/L to final concentrations of 8.9 ± 5.8 nmol/L. Despite this substantial relative increase, neither serum 25(OH)D_3_ nor total 25(OH)D changed significantly. Conversely, vitamin D_3_ supplementation elevated serum 25(OH)D_3_ by 55% from a baseline of 44.0 ± 17.1 nmol/L, leading to significantly higher total 25(OH)D levels compared with placebo (57.3 ± 17.7 vs. 41.7 ± 20.1 nmol/L). An additional observation was a reduction in plasminogen activator inhibitor-1 following vitamin D_2_ intake, suggesting a possible effect on fibrinolytic pathways; however, this did not translate into improved vitamin D status. The authors concluded that each isoform increased its respective hydroxylated metabolite, but only vitamin D_3_ produced a clinically meaningful improvement in overall vitamin D concentrations [48].

A recent and comprehensive systematic review with meta-analysis of randomized controlled trials provides important insight into the metabolic differences between vitamin D_2_ and vitamin D_3_. The analysis, covering studies published between 1975 and 2023, evaluated the impact of vitamin D_2_ supplementation on circulating concentrations of 25-hydroxyvitamin D_3_ [25(OH)D_3_] [49]. The pooled results demonstrate that individuals receiving vitamin D_2_ experienced a significant decline in serum 25(OH)D_3_ compared with non-supplemented controls. This reduction was observed both in end-of-intervention comparisons between groups and in analyses of changes from baseline. These findings suggest that vitamin D_2_ supplementation does not simply add to total vitamin D status but may also influence the metabolism or clearance of the D_3_-derived metabolite. Several mechanisms may explain this interaction. Available evidence suggests that the lower efficacy of vitamin D_2_ is unlikely to result mainly from poorer intestinal absorption, as vitamin D_2_ and vitamin D_3_ appear to be absorbed with similar efficiency in human studies and intestinal cell models [50]. Therefore, the key differences seem to occur after the absorption, particularly during transport, hydroxylation, and catabolism [50]. The structural features of vitamin D_2_, including a double bond between C22 and C23 and a methyl group at C24, reduce the affinity of vitamin D-binding protein (DBP) for vitamin D_2_ and its metabolites compared with the corresponding D_3_ forms [6,7]. Because DBP maintains the circulating reservoir of 25(OH)D and prolongs its half-life, weaker binding of 25(OH)D2 may increase the fraction available for metabolism and clearance, thereby contributing to faster removal from the circulation [6,7]. Differences are also observed at the enzymatic level. The hepatic 25-hydroxylase CYP2R1 hydroxylates both vitamins comparably, whereas the mitochondrial CYP27A1 does not 25-hydroxylate vitamin D_2_, and the catabolic 24-hydroxylase CYP24A1 processes 1,25(OH)_2_D_2_ differently from 1,25(OH)_2_D_3_ [6]. Together, these post-absorptive differences may help explain why vitamin D_2_ often produces a smaller and less sustained increase in circulating 25(OH)D than equivalent doses of vitamin D_3_, particularly with intermittent or bolus dosing regimens [6]. The observed inverse association between D_2_ and D_3_ metabolites further supports the concept that these two forms are not metabolically equivalent and raises the questions about their full interchangeability in supplementation strategies [49].

Overall, the available studies suggest that vitamin D_2_ effectively increases circulating 25(OH)D_2_, but its effect on total 25(OH)D is often less consistent than that of vitamin D_3_. In food-based interventions, this response may be further influenced by the food matrix and processing conditions, including cooking losses in mushroom products, which may limit the bioefficacy of vitamin D_2_.

The evidence favoring vitamin D_3_ is not uniform, however, and should be interpreted cautiously. Several trials found the two forms comparable, particularly at low daily doses: similar effects on total 25(OH)D were reported at 1000 IU/day in healthy adults [36,37], at 400 IU/day in breastfed infants [35], and at 200–400 IU/day, where the two forms appeared equipotent [45]. An exception was observed at a fortification dose of 600 IU/day, where vitamin D_3_ raised total 25(OH)D by about 75% compared with 33% for vitamin D_2_ in a large trial [46]. In contrast, the advantage of vitamin D_3_ was clearest with higher-dose regimens, including 2000 IU/day [39], 50,000 IU/week [41], and a single dose of 300,000 IU [44]. Overall, this suggests a dose-dependent tendency, although not the absolute one. Interpretation is also limited by small sample sizes, short intervention periods, unequal group sizes, and heterogeneous analytical methods, including LC-MS/MS, HPLC, RP-HPLC, RIA, and CLIA. These factors call for a cautious interpretation of the apparent superiority of vitamin D_3_.

### 3.5. Effectiveness of Vitamin D_2_ Supplementation in Special Groups of Patients

#### 3.5.1. Chronic Kidney Diseases

Wetmore et al. [51], in a randomized clinical study, compared weekly administration of vitamin D_2_ (1250 μg, 50,000 IU) with an equivalent dose of vitamin D_3_ in non-dialysis-dependent patients with stage 3–5 CKD over a 12-week treatment period. Vitamin D_2_ ingestion resulted in a substantial rise in total serum 25(OH)D during active treatment, confirming its ability to correct vitamin D deficiency in CKD patients. However, the magnitude of increase achieved (30.7 ng/mL) was smaller than that observed with vitamin D_3_ (45.0 ng/mL) at the end of the intervention period of 12 weeks. This difference was largely attributable to a decline in the endogenous 25(OH)D_3_ fraction during vitamin D_2_ therapy, which limited the net increase in total 25(OH)D. Following discontinuation of supplementation, serum 25(OH)D concentrations declined in both treatment groups. Importantly, six weeks after cessation of therapy, no statistically significant difference in total 25(OH)D levels was observed between patients previously treated with ergocalciferol and those receiving cholecalciferol. This finding suggests that, despite the lower peak responses during active treatment, vitamin D_2_ and vitamin D_3_ may show comparable short-term persistence once supplementation is stopped. No significant differences were detected between both vitamins with respect to changes in serum parathyroid hormone or circulating 1,25-dihydroxyvitamin D, indicating that both forms exert similar effects on downstream markers of mineral metabolism in non-dialysis-dependent CKD patients. These findings demonstrate that vitamin D_3_ can be more effective in increasing serum 25(OH)D concentrations in CKD during active supplementation.

#### 3.5.2. Insulin Related Diseases

In a 12-week double-blind RCT vitamin D_2_ (50,000 IU) was administered weekly to 90 healthy adults with low vitamin D level in serum (<20 ng/mL of 25(OH)D), to evaluate its impact on glucose and insulin metabolism. Total 25(OH)D increased significantly from 18 ± 7 to 43 ± 12 ng/mL, but insulin secretion and sensitivity were unchanged. Vitamin D_2_ corrected the deficiency but did not necessarily improve metabolic markers [52]. A similar study compared the supplementation of weekly administered 20,000 IU vitamin D_2_ or 15,000 IU vitamin D_3_ for 3 months to 47 Thai adults with impaired glucose regulation. The treatment significantly increased total 25(OH)D, and the oral glucose tolerance test, insulin resistance (HOMA-IR) and insulin secretion index (HOMA%B) were calculated. After the intervention, the oral glucose tolerance status did not change significantly and did not differ between groups, while HOMA-IR and HOMA%B showed no significant change in the overall vitamin D group. However, a significant reduction in HOMA-IR (−0.24 ± 0.42), together with an increased disposition index (+5.1 ± 10.5), was observed only in the subgroup whose total 25(OH)D rose by ≥10 ng/mL. Vitamin D_3_ raised 25(OH)D_3_ by +13.7 ± 4.9 ng/mL, whereas vitamin D_2_ increased 25(OH)D_2_ by +25.9 ± 4.2 ng/mL but reduced 25(OH)D_3_ by −13.1 ± 3.1 ng/mL. All changes were significant. Overall, vitamin D supplementation produced only limited metabolic benefits, mainly a reduction in waist circumference, with an improvement in insulin resistance confined to participants achieving an adequate rise in 25(OH)D, while vitamin D_3_ may exert stronger anthropometric effects [53].

#### 3.5.3. Burn Injury

A randomized, double-blind study evaluated the effects of daily vitamin D_2_ or D_3_ supplementation (100 IU/kg) in 50 pediatric patients with severe burns (mean total body surface area 55.7% ± 2.6%). Serum vitamin D markers and parathyroid hormone (PTH) were monitored from hospitalization to one year post-injury. Although no significant differences in vitamin D levels were observed between groups during hospitalization, deficiency remained common at discharge (>10%) and increased markedly after one year, affecting 75% of the placebo group, 56% of the vitamin D_2_ group, and 25% of the vitamin D_3_ group. While changes in PTH and clinical outcomes were not statistically significant, supplementation showed clinically meaningful trends toward reduced insulin requirements, sepsis, and scar formation. These findings suggest prolonged vitamin D impairment after severe burns and support extended vitamin D_3_ supplementation to improve long-term status [54].

### 3.6. Effectiveness of Vitamin D_2_-Rich Mushrooms’ Supplementation in Special Groups of Patients

A 6-month randomized, double-blind, placebo-controlled trial evaluated the effects of vitamin D_3_ (~600 IU/day), vitamin D_2_ delivered in a mushroom matrix, standard mushrooms, and placebo, on vitamin D status, cognition, and mood in 436 healthy adults aged ≥60 years. The mushroom species used in the intervention was not reported in the original publication. Total 25(OH)D and 25(OH)D_3_ concentrations were maintained only in the vitamin D_3_ group, whereas significant declines were observed in the vitamin D_2_, mushroom, and placebo arms, with responses influenced by baseline vitamin D levels. Despite observational links between vitamin D deficiency and cognitive decline, no improvements in cognitive performance or mood were detected in any intervention group. These findings indicate that moderate-dose vitamin D_3_ is effective for maintaining vitamin D status during winter, but supplementation does not confer measurable cognitive or mood benefits in healthy older adults [55].

Another randomized, double-blind, placebo-controlled trial evaluated whether supplementation with vitamin D_2_ derived from Portobello mushroom (*Agaricus bisporus*) powder could improve skeletal muscle function and attenuate exercise-induced muscle damage in young athletes with low vitamin D status. The study included 33 male high school athletes with baseline serum 25(OH)D < 30 ng/mL. Participants were randomly assigned to receive capsules containing vitamin D_2_ (600 IU/day) from UV-exposed Portobello mushrooms, or the placebo containing non-UV-exposed mushroom powder, for 6 weeks. Following supplementation, participants completed a 90 min exercise protocol designed to induce skeletal muscle damage. Biochemical markers and measures of muscle function were assessed at baseline, post-supplementation (pre-exercise), and post-exercise. Vitamin D_2_ supplementation resulted in a significant increase in serum 25(OH)D_2_ (approximately 9–10 fold), accompanied by a significant decrease in serum 25(OH)D_3_ (~28%) and a modest increase in total 25(OH)D. Despite these changes, no significant differences were observed between groups in muscle strength, markers of muscle damage (myoglobin, LDH, AST, creatine kinase), or delayed onset muscle soreness. It was concluded that 6-week supplementation with vitamin D_2_ from Portobello mushrooms effectively increased circulating 25(OH)D_2_ but did not improve muscle function or protect against exercise-induced muscle damage in young athletes with low baseline vitamin D status [56].

Mehrotra et al. [57] conducted a 16-week randomized feeding trial to evaluate whether daily consumption of UVB-treated mushrooms enriched with vitamin D_2_ improves vitamin D status and metabolic risk factors in vitamin D-deficient adults with prediabetes and BMI > 25. The study used fresh market-available mushrooms, but the species was not indicated. Forty-three participants were assigned to consume entrées containing mushrooms providing either 600 IU or 4000 IU of vitamin D_2_, or untreated mushrooms combined with vitamin D_3_ supplements at comparable labeled doses. Due to substantial cooking losses, the effective vitamin D_2_ intake was lower than expected and resulted in only modest or no increases in serum 25(OH)D_2_ and total 25(OH)D compared with the vitamin D_3_ groups, whose actual intake exceeded labeled amounts. No intervention modified risk factors associated with type 2 diabetes. The limited response to mushroom-derived vitamin D_2_ may reflect reduced stability during cooking, as well as lower absorption and/or hydroxylation in this overweight, prediabetic population.

Notably, this study highlights important practical limitations of food-based vitamin D_2_ delivery. Thermal processing substantially reduced the vitamin D_2_ content of UVB-treated mushrooms, suggesting that postharvest fortification strategies may not guarantee predictable intake under real-world culinary conditions. Furthermore, the weaker response observed in overweight, prediabetic individuals may indicate altered vitamin D metabolism in metabolically compromised populations, potentially related to sequestration in adipose tissue or impaired hydroxylation. Collectively, these findings raise concerns about the reliability of mushroom-derived vitamin D_2_ as a strategy for improving vitamin D status in high-risk groups and underscore the need to optimize food fortification approaches.

Nieman et al. [58] investigated the physiological consequences of vitamin D_2_ supplementation in a cohort of athletes. In this double-blind randomized placebo-controlled study, 30 NASCAR pit crew members were assigned to receive either placebo (*n* = 15) or 3800 IU/day of vitamin D_2_ derived from UV-treated Portobello mushroom powder (*n* = 13) for six weeks. Supplementation produced a 456% increase in serum 25(OH)D_2_, accompanied by a 21% decline in 25(OH)D_3_, yet total vitamin D concentrations did not change significantly. Functional outcomes were similarly unaffected, as no improvements were detected in strength or performance tests. Unexpectedly, biomarkers of exercise-induced muscle damage were higher in the vitamin D_2_ group following an eccentric training protocol, with myoglobin rising by 252% compared with 122% in the placebo group (*p* = 0.001) and creatine phosphokinase increasing by 169% versus 32% (*p* < 0.001). These findings suggest a possible interaction between vitamin D isoforms, where elevation of 25(OH)D_2_ may occur at the expense of circulating 25(OH)D_3_, although the clinical relevance of this shift remains uncertain. D_2_ alters metabolite balance and may negatively influence exercise-induced muscle damage markers.

### 3.7. Effectiveness of Algal Supplementation in Special Groups of Patients

Despite growing interest in algae as a potential plant-based source of vitamin D, there are currently no randomized controlled trials (RCTs) demonstrating that supplementation with natural algal biomass (e.g., *Ulva*, *Chlorella*, *Spirulina*, or other microalgae) increases circulating 25-hydroxyvitamin D [25(OH)D] concentrations in humans. In contrast, human intervention studies involving algae have largely focused on other bioactive components. For example, Vanlint and Ried [59] conducted a pilot RCT evaluating algal oil supplementation for bone health; however, vitamin D_3_ (1000 IU/day) was administered separately and not derived from algae. Another study examined supplementation with *Phaeodactylum tricornutum* in elderly individuals but assessed antioxidant and metabolic outcomes rather than vitamin D status [60]. Numerous trials involving *Chlorella*, *Spirulina*, and other algal products have similarly evaluated lipid metabolism, inflammatory markers, or immune responses without measuring serum 25(OH)D.

While experimental studies confirm that certain algae can contain vitamin D under UV exposure, no clinical trials have demonstrated that algal supplementation increases serum 25(OH)D concentrations in humans. The lack of standardized preparations, variable vitamin D content, and absence of bioavailability studies represent significant research gaps that must be addressed before algae can be considered a validated dietary source of vitamin D. Only one prospective eight-month study exists, conducted by Schwarz et al. [61], with a group of vegans adhering to a whole-food, unrefined, organic diet without dietary supplementation. Participants consumed a minimum of 12 g/week of nori algae and 15 g/week of sun-dried wild mushrooms. While the primary nutritional focus of this group concerned vitamin B_12_ intake, serum vitamin D_2_ and D_3_ concentrations were also longitudinally assessed. At baseline, serum vitamin D_3_ concentrations did not differ significantly between groups; values in vegan participants were within the grey area (30–50 nmol/L), whereas vegetarians and meat-eaters generally presented normal levels. Over the course of the study, vitamin D_3_ concentrations declined significantly in the vegan group. By the end of the study, vitamin D_3_ levels in this group were below the reference threshold and significantly lower than in supplemented vegans and vegetarians. Thus, regular consumption of nori algae and sun-dried mushrooms did not prevent a deterioration in vitamin D_3_ status. Importantly, the study does not demonstrate that nori algae itself was the primary contributor to the observed vitamin D_2_ increase. The findings indicate that inclusion of nori algae within a whole-food vegan diet does not ensure maintenance of adequate vitamin D levels, particularly with respect to vitamin D_3_, and that dietary sources alone may be insufficient to meet physiological requirements.

The absence of clinical evidence may be explained by several factors. First, reported vitamin D concentrations in algae are highly variable and strongly dependent on UV exposure, species differences, and cultivation conditions, which complicates standardization. Second, most commercially available algal supplements are not standardized for vitamin D content, making dose control difficult. Third, vitamin D formation in algae appears to be largely photochemical rather than enzymatically regulated, raising questions about stability and reproducibility. Finally, the bioavailability of algal-derived vitamin D_2_ or D_3_ has not been systematically investigated, and its metabolic conversion to 25(OH)D in humans remains unverified.

## 4. Limitations

Several limitations of the present narrative review should be acknowledged. First, although the literature search was structured and supported by a PRISMA-style flow diagram, the manuscript was designed as a narrative review rather than a systematic review or meta-analysis. Therefore, no formal risk-of-bias assessment, quality scoring system, or quantitative meta-analysis was performed. This limits the possibility of drawing pooled estimates regarding the comparative effectiveness of vitamin D_2_ and vitamin D_3_.

Second, the search was conducted in PubMed, ScienceDirect, and Google Scholar, which were selected to provide broad coverage of biomedical, nutritional, food science, biochemical, analytical, and clinical studies. However, the Cochrane Central Register of Controlled Trials/CENTRAL was not included as a primary search database. Since CENTRAL is particularly relevant for identifying randomized controlled trials, some clinical trials directly comparing vitamin D_2_ and vitamin D_3_ may not have been captured. Consequently, conclusions regarding comparative clinical potency should be interpreted cautiously and within the narrative scope of this review.

Third, the evidence included in this review is heterogeneous. Studies on edible mushrooms and algae mainly include analytical, food composition, technological, and experimental investigations, whereas the comparison of vitamin D_2_ and vitamin D_3_ is based primarily on human intervention studies. These different evidence streams cannot be directly compared in terms of methodological design, sample size, outcome measures, or strength of inference. In particular, data on vitamin D content in mushrooms, macroalgae, and microalgae are strongly influenced by species, growth conditions, UV exposure, moisture content, analytical method, storage, and culinary processing.

Fourth, the available human studies comparing vitamin D_2_ and vitamin D_3_ differ substantially in population characteristics, baseline vitamin D status, age, health condition, dose, dosing frequency, route of administration, duration of intervention, and analytical methods used to measure vitamin D metabolites. Some trials included small sample sizes, short intervention periods, or unequal group sizes, which may limit the generalizability of their findings. Moreover, studies used different analytical techniques, including LC-MS/MS, HPLC, RP-HPLC, RIA, and CLIA, which may contribute to variability in reported serum 25(OH)D_2_, 25(OH)D_3_, and total 25(OH)D concentrations.

Fifth, the geographical distribution of the included studies was uneven. Human intervention studies were concentrated mainly in a limited number of countries, particularly the USA, while mushroom, algae, microalgae, and seaweed studies were distributed across several countries but often represented by only one or two studies per country (Supplementary Figure S1). This geographical imbalance may limit the global representativeness of the conclusions, especially because vitamin D status, UV exposure, dietary habits, supplement use, food fortification practices, and availability of plant-based vitamin D sources vary substantially between regions.

Sixth, the literature covered a broad time span from 1996 to 2026. As shown in Supplementary Figure S2, publication activity was relatively limited in the earlier years but increased markedly in more recent years, reflecting growing interest in vitamin D_2_ sources, UV-mediated formation, algae/microalgae, and comparison between vitamin D_2_ and vitamin D_3_. Over this period, analytical methods for vitamin D determination evolved substantially, particularly with the increasing use of LC-MS/MS-based approaches. As a result, older studies may not be fully comparable with more recent investigations using more sensitive and selective analytical techniques. This limitation is particularly relevant for algae and microalgae, where vitamin D concentrations are often low and may be affected by analytical sensitivity, co-elution, and interference from lipophilic pigments.

Finally, evidence concerning algae as a dietary source of vitamin D remains especially limited. Although several analytical studies suggest that selected macroalgae and microalgae may contain vitamin D_2_ or vitamin D_3_ after UV exposure, there is currently insufficient human intervention evidence demonstrating that natural algal biomass reliably improves circulating 25(OH)D concentrations. Therefore, conclusions regarding algae as a functional or clinically relevant vitamin D source should be considered preliminary. 

## 5. Conclusions

The growing interest in plant-based and sustainable sources of vitamin D has intensified research into mushrooms and algae as alternative providers of vitamin D_2_ and, under specific conditions, vitamin D_3_. Evidence clearly demonstrates that UV exposure effectively enhances vitamin D_2_ content in edible mushrooms, making them one of the few non-animal dietary sources capable of delivering nutritionally relevant amounts of this vitamin. However, technological factors such as processing, storage, and cooking may substantially reduce the final vitamin D_2_ intake.

The current data on algae remains inconsistent and largely preliminary. Although certain micro- and macroalgal species can contain vitamin D following UV exposure, there is no robust clinical evidence demonstrating that algal supplementation improves circulating 25(OH)D concentrations in humans. Variability in sterol composition, lack of standardization, and insufficient bioavailability represent major limitations.

Human intervention trials consistently indicate that vitamin D_2_ increases circulating 25(OH)D_2_ but often reduces endogenous 25(OH)D_3_ and is generally less effective than vitamin D_3_ in raising total vitamin D status. Recent meta-analytic evidence further supports the concept that vitamins D_2_ and D_3_ are not metabolically equivalent [49]. Available clinical data suggest that while vitamin D_2_ may represent a suitable non-animal source for individuals following plant-based diets, vitamin D_3_ demonstrates greater efficacy in elevating and maintaining circulating vitamin D concentrations. These observations have direct practical implications. In food fortification, vitamin D_3_ achieves substantially greater improvements in vitamin D status than equivalent doses of vitamin D_2_, particularly in populations at higher risk of deficiency [46], although the two forms appear more comparable at low dietary doses [45]. For vegan and vegetarian consumers, for whom vitamin D_2_ derived from UV-treated mushrooms, fermented yeast, or ergosterol synthesis remains the principal non-animal option, its lower potency and shorter duration of action should be reflected in dosing recommendations [33], and its dietary contribution interpreted in light of the considerable losses that occur during processing and cooking [57].

Future research should focus on mechanistic clarification of D_2_ and D_3_ interactions, optimization of food fortification strategies and production, and well-designed long-term clinical trials evaluating functional plant-based vitamin D sources.

## Figures and Tables

**Table 2:** Losses of vitamin D_2_ during storage and culinary processing of mushrooms.

Process	Vitamin D_2_ Loss	Reference
Refrigerated storage (fresh), 2–4 °C	Minimal over 7–14 days; gradual first-order decline thereafter	[18,19,20]
Dry storage (dried)	14.3 → 9.3 µg/g DM over 8 months; → 6.9 µg/g DM over the next 10 months	[11]
Frying without oil (~5 min)	12–15%	[21,22]
Boiling	~40%	[21,22]
Oven-baking	~40%	[21,22]

**Table 3:** Reported vitamin D_2_ and vitamin D_3_ contents of micro- and macroalgae across the cited studies.

Algal Group	Species	Treatment/Condition	Vitamin D_2_ Content	Vitamin D_3_ Content	Analytical Method	Reference /Country
Microalgae	(*Pediastrum*, *Scenedesmus*, *Crucigenia*, *Coelastrum*, *Chlorella*, *Cosmarium*), (*Gomphosphaeria*, *Oscillatoria*), diatoms (*Gomphonema*, *Synedra*, *Navicula*, *Cyclotella*), *Euglena*	Wild-harvested (lake); summer (April)	5.3 µg/100 g DW	80 µg/100 g DW	HPLC	[24] India
Microalgae	*Nannochloropsis*-like sp., *Pavlova pinguis*, *Stichococcus* sp., *Tetraselmis* sp.	Cultured; fluorescent light; late-log harvest	Below detection (≤0.45 µg/g)	Below detection (≤0.45 µg/g)	HPLC	[25] Australia
Microalga	*Nannochloropsis oceanica*	Cultured; UVB (312 nm); dose-dependent, up to 36 kJ/m^2^/day (lamp 5–15 cm; max at 5 cm); 5 days; 23 ± 1 °C	Up to 0.27 ± 0.08 µg/g DW	Up to 1 ± 0.3 µg/g DW	LC-MS/MS	[26] Denmark
Microalgae	*Chlorella minutissima*, *Arthrospira maxima*, *Rhodomonas salina*	Cultured; UVB (312 nm); 3–22 kJ/m^2^/day (lamp 10 cm); 7 days; 23 ± 1 °C	Below LOQ (*R. salina* up to 0.20 µg/g DW)	Not produced (below LOQ)	LC-MS/MS	[26] Denmark
Microalga	*Emiliania huxleyi*	Cultured; full-spectrum UV (UV-A 0.50, UV-B 0.07, UV-C 0.03 W/m^2^; peaks 355/297/265 nm); lamp 20 cm; 14 h/day (diurnal); 18 °C	4.32 ± 1.39 ng/mg DW	0.038 ± 0.001 ng/mg DW	UPC^2^-MS/MS	[28] Israel
Microalga	*Emiliania huxleyi*	Cultured; no UV (control); 18 °C	0.09 ± 0.01 ng/mg DW	0.039 ± 0.001 ng/mg DW	UPC^2^-MS/MS	[28] Israel
Microalga	*Nannochloropsis limnetica*	Cultured; UVB; 15 kJ/m^2^/day; 3 days	-	2700 ± 198 ng/g DW	HPLC	[29] Denmark
Macroalgae	Wakame (*Undaria pinnatifida*), kombu (*Lessonia corrugata*)	Wild-harvested, winter	Not detected (<0.05 µg/100 g DW)	Not detected (<0.05 µg/100 g DW)	LC-QQQ	[30] Australia

DW, dry weight; HPLC, high-performance liquid chromatography; LC-MS/MS, liquid chromatography–tandem mass spectrometry; LC-QQQ, liquid chromatography–triple quadrupole mass spectrometry; LOQ, limit of quantification; UPC^2^-MS/MS, ultra-performance convergence (supercritical fluid) chromatography–tandem mass spectrometry; UV, ultraviolet.

**Table 4:** Summary of intervention studies comparing vitamin D_2_ and vitamin D_3_ on circulating vitamin D metabolite concentrations.

Reference/Type of Study/Country	Population	Intervention	Analytical Method	Main Outcomes
Nimitphong et al. [34] RCT, unblinded; Thailand	*n* = 39 healthy adults; 15–70 y; 61.5% vitamin D deficient (<50 nmol/L)	D_2_ or D_3_ 400 IU/day; daily; 3 months; tablets (+calcium)	LC-MS/MS	D_3_: 25(OH)D_3_ +16.2 ± 4.2 nmol/L (*p* < 0.001). D_2_: 25(OH)D_2_ +22.0 ± 2.1 nmol/L (*p* < 0.001) but 25(OH)D_3_ −14.2 nmol/L (*p* < 0.001). Total 25(OH)D tended higher with D_3_ (67.8 vs. 61.0 nmol/L; *p* = 0.08).
Gallo et al. [35] RCT; Canada	*n* = 52 healthy breastfed infants; 1 month; 23% vitamin D deficient (≤24.9 nmol/L)	D_2_ or D_3_ 400 IU/day; daily; 3 months; drops	LC-MS/MS; CLIA	No difference in total 25(OH)D (D_2_ +17.6 vs. D_3_ +22.2 nmol/L; *p* = 0.21). Sufficiency ≥50 nmol/L: D_3_ 96% vs. D_2_ 75% (*p* = 0.05).
Holick et al. [36] RCT, double blind, placebo controlled; USA	*n* = 68 healthy adults; 18–84 y; 60% vitamin D deficient (<20 ng/mL)	D_2_ 1000, D_3_ 1000, or D_2_ + D_3_ 500 + 500 IU/day, or placebo; daily; 11 weeks; capsules	LC-MS/MS	Total 25(OH)D: D_2_ 16.9→26.8 ng/mL (*p* = 0.023); D_3_ 19.6→28.9 ng/mL (*p* = 0.027); D_2_ + D_3_ 20.2→28.4 ng/mL (*p* = 0.041). No difference between active groups (*p* = 0.957).
Biancuzzo et al. [37] RCT, double blind, placebo controlled; USA	*n* = 34 healthy adults; 18–79 y; 82% vitamin D insufficient (<30 ng/mL)	D_2_ or D_3_ 1000 IU/day, or placebo; daily; 11 weeks; capsules or orange juice	LC-MS/MS	Both forms raised total 25(OH)D similarly. D_2_: 1,25(OH)D_2_ + 7.4 pg/mL and 1,25(OH) D_3_ −9.9 pg/mL (total active unchanged).
Glendenning et al. [38] RCT, double blind; Australia	*n* = 95 hip-fracture patients; elderly (mean ~83 y); vitamin D insufficient (<50 nmol/L)	D_2_ or D_3_ 1000 IU/day; daily; 3 months; tablets/capsules (+calcium carbonate 600 mg/day)	HPLC; RIA	D_3_ raised 25(OH)D more than D_2_: +31% by HPLC (*p* = 0.010); +52% by RIA (*p* < 0.001).
Lehmann et al. [39] RCT, double blind, placebo controlled; Germany	*n* = 107 healthy adults; 19–67 y	D_2_ or D_3_ 2000 IU/day, or placebo; daily; 8 weeks; tablets	LC-MS/MS	Total 25(OH)D at 8 wk: D_3_ +45.5 vs. D_2_ +30.2 nmol/L (*p* = 0.001). 25(OH)D_3_: D_3_ +46.5 vs. D_2_ −19.8 nmol/L (*p* = 0.001).
Trang et al. [40] RCT, double blind; Canada	*n* = 72 adults (D_2_ 17; D_3_ 55); 38 ± 9 y	D_2_ or D_3_ 4000 IU/day; daily; 14 days; oral solution	RIA	Increase in 25(OH)D: D_3_ +23.3 vs. D_2_ +13.7 nmol/L (*p* = 0.03).
Heaney et al. [41] RCT, single-blind; USA	*n* = 33 healthy adults; 49.5 ± 9.8 y; baseline status D_2_ 30.6 ± 14.8; D_3_ 26.0 ± 9.2 ng/mL	D_2_ or D_3_ 50,000 IU/week; weekly; 12 weeks; gel capsules	CLIA; HPLC	25(OH)D incremental AUC (12 wk): D_3_ 2136 ± 606 vs. D_2_ 1366 ± 516 ng·d/mL (*p* < 0.005). Steady-state increment: D_3_ +45 ± 16.2 vs. D_2_ +24 ± 10.3 ng/mL (*p* < 0.001).
Binkley et al. [42] RCT, double blind; USA	*n* = 64 older adults; 65–88 y; 40% with 25(OH)D < 30 ng/mL	D_2_ or D_3_ 1600 IU/day or 50,000 IU/month; daily or monthly; 12 months; capsules	RP-HPLC	D_3_ > D_2_ at 12 mo: daily +9.2 vs. +6.1 ng/mL (*p* = 0.05); monthly +8.9 vs. +3.6 ng/mL (*p* = 0.11).
Hammami et al. [43] RCT, double blind, placebo controlled; Saudi Arabia	*n* = 100 healthy adults; ≥18 y;	D_2_ or D_3_ 50,000 IU single oral dose, or placebo; followed 56 days; soft gel capsules	RP-HPLC	D_2_ → 25(OH)D_3_ vs. placebo: −13.2 nmol/L day 28 (*p* < 0.001); −10.8 nmol/L day 56 (*p* < 0.001). D_3_ → 25(OH)D_2_ vs. placebo: −9.8 nmol/L day 28 (*p* < 0.001); −1.7 nmol/L day 56 (*p* = 0.71).
Romagnoli et al. [44] RCT; Italy	*n* = 32 elderly women; 66–97 y; all vitamin D deficient	D_2_ or D_3_ 300,000 IU single dose, by oral (os) or intramuscular (im) route;	RIA	30-day rise in 25(OH)D: D_3_ os +47.8 ± 7.3 vs. D_3_ im +15.9 ± 11.3, D_2_ os +17.3 ± 4.7, D_2_ im +5.0 ± 4.4 ng/mL (all *p* < 0.001). AUC_60_: D_3_ os 3193 ± 759 vs. D_2_ os 1820 ± 512 ng d/mL (*p* < 0.001); D_3_ im 1361 ± 492 vs. D_2_ im 728 ± 195 (*p* < 0.01).

LC-MS/MS, liquid chromatography–tandem mass spectrometry; HPLC, high-performance liquid chromatography; RP-HPLC, reversed-phase high-performance liquid chromatography; RIA, radioimmunoassay; CLIA, chemiluminescence immunoassay; 25(OH)D, 25-hydroxyvitamin D; 1,25(OH)D_2_, 1,25-dihydroxyvitamin D; IU, international units.

## Data Availability

No new data were created or analyzed in this study. Data sharing is not applicable to this article.

## Supplementary Materials

The following supporting information can be downloaded at https://www.mdpi.com/article/10.3390/metabo16070485/s1, Publication Trends and Geographical Distribution of Included Studies. Figure S1. Geographical distribution of included studies according to study type (PubMed). Figure S2. Number of publications by year (PubMed).

## Abbreviations

The following abbreviations are used in this manuscript:

25(OH)D	25-hydroxyvitamin D
25(OH)D_2_	25-hydroxyvitamin D_2_ (25-hydroxyergocalciferol)
25(OH)D_3_	25-hydroxyvitamin D_3_ (25-hydroxycholecalciferol)
1,25(OH)_2_D	1,25-dihydroxyvitamin D (calcitriol; total)
1,25(OH)_2_D_2_	1,25-dihydroxyvitamin D_2_
1,25(OH)_2_D_3_	1,25-dihydroxyvitamin D_3_
AUC	Area Under the Curve
BMI	Body Mass Index
CKD	chronic kidney disease
DBP	Vitamin D-Binding Protein
DM	Dry Mass
EFSA	European Food Safety Authority
EMA	European Medicines Agency
FDA	U.S. Food and Drug Administration
FW	Fresh Weight
HOMA-IR	Homeostatic Model Assessment of Insulin Resistance
HOMA%B	Homeostatic Model Assessment of β-cell Function
IU	International Unit(s)
LC–QQQ	Liquid Chromatography–Triple Quadrupole Mass Spectrometry
PTH	Parathyroid Hormone
RCT	Randomized Controlled Trial
RIA	Radioimmunoassay
UV-A/UV-B/UV-C	Ultraviolet A/B/C
