# Supplementary material for: Content of Vitamin D2 in Alternative Biological and Nutritional Sources and Its Effectiveness as Compared to Vitamin D3—A Narrative Review
Source: Metabolites. 2026 Jul 10;16(7):485. doi: 10.3390/metabo16070485 (PMC13414236; doi:10.3390/metabo16070485)
Supplement: Supplementary file 1 [file metabolites-16-00485-s001.zip › metabolites-4373620-supplementary.pdf]

**Supplementary Material: Publication Trends and Geographical Distribution of Included Studies**

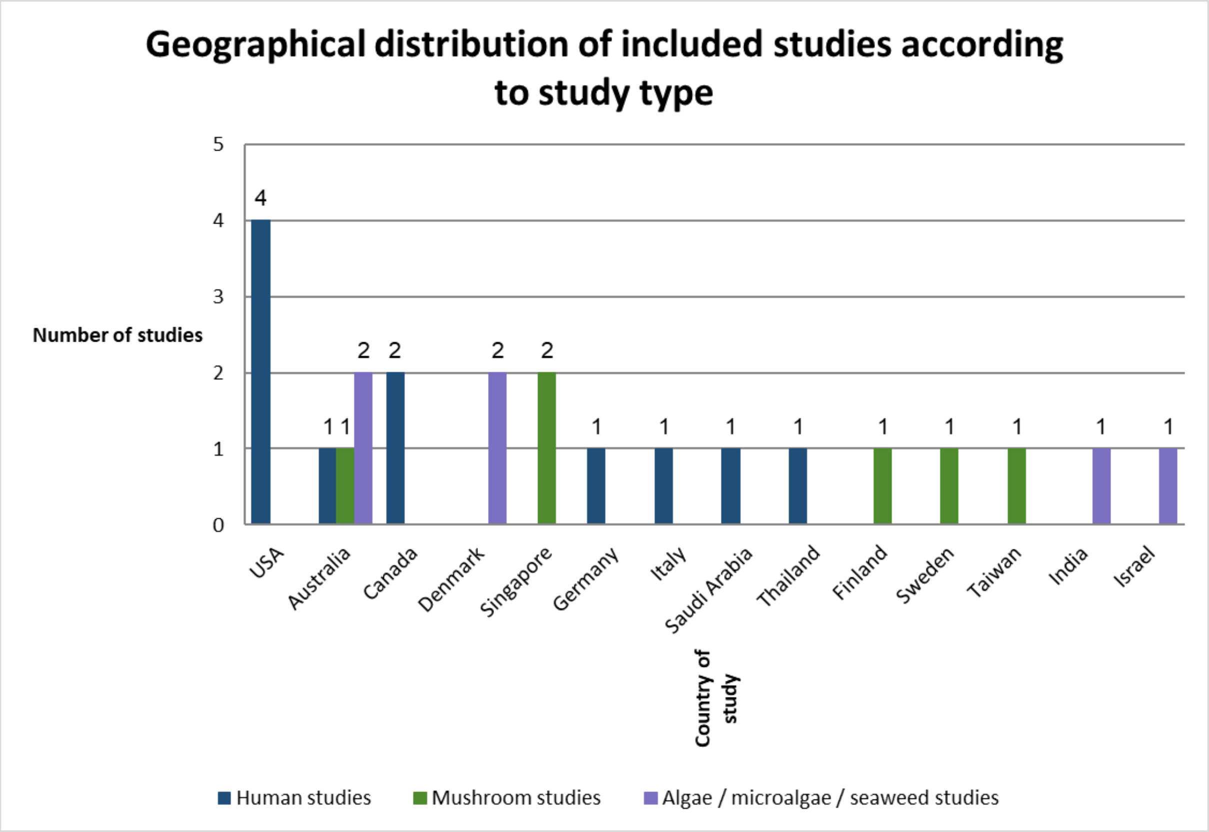


**Figure S1. Geographical distribution of included studies according to study type (PubMed).**

The figure presents the geographical distribution of studies included in the review, classified into human studies, mushroom studies, and algae/microalgae/seaweed studies. The distribution shows that human intervention studies were most frequently represented by studies from the USA, followed by individual or smaller numbers of studies from countries such as Australia, Canada, Germany, Italy, Saudi Arabia, and Thailand. Mushroom studies were reported from several countries, including Australia, Singapore, Finland, Sweden, and Taiwan, whereas algae, microalgae, and seaweed studies were distributed across countries such as Australia, Canada, Denmark, India, and Israel. This distribution highlights the geographical heterogeneity of the available evidence and indicates that some regions and study types are represented by only a limited number of publications.

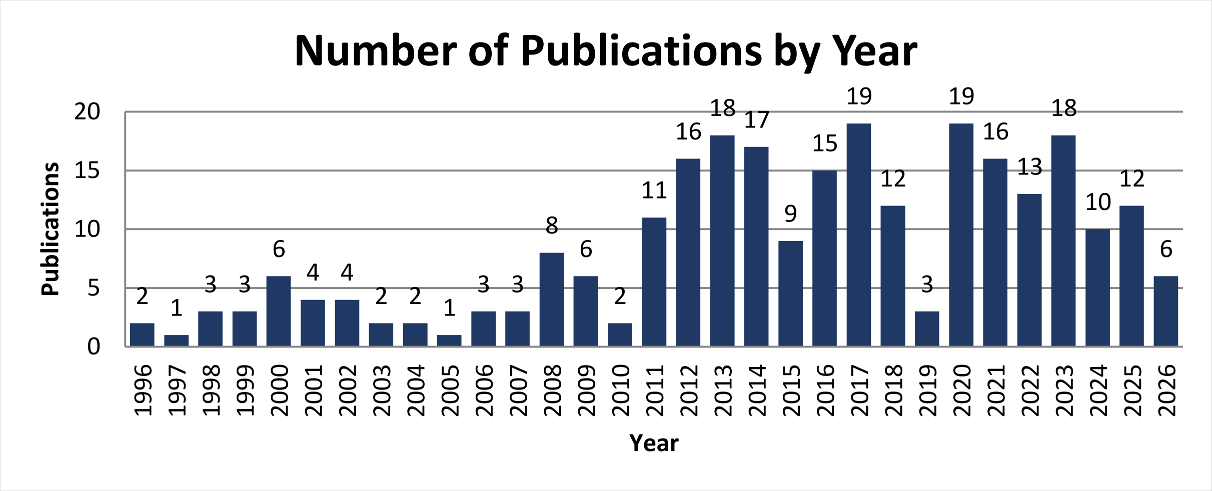


Search query: ("vitamin D2"[tiab] OR ergocalciferol[tiab]) AND (mushroom*[tiab] OR algae[tiab] OR microalga*[tiab] OR seaweed[tiab] OR "dietary supplement*"[tiab] OR "pharmaceutical formulation*"[tiab] OR pharmac*[tiab] OR "food science"[tiab] OR ultraviolet[tiab] OR UVB[tiab] OR irradiat*[tiab])

**Figure S2. Number of publications by year (PubMed).**

The figure illustrates the annual number of publications identified using the applied search query covering vitamin $D_2$/ergocalciferol in relation to mushrooms, algae, microalgae, seaweed, dietary supplements, pharmaceutical formulations, food science, ultraviolet exposure, UVB, and irradiation. The publication trend indicates relatively limited research activity before 2010, followed by a marked increase in the number of publications from approximately 2012 onward. Peaks are observed in several recent years, reflecting growing scientific interest in alternative vitamin D sources, UV-mediated vitamin D formation in biological matrices, and the comparative relevance of vitamin $D_2$ and vitamin $D_3$ in nutrition and supplementation.
